# *Calycophyllum spruceanum* (Benth.), the Amazonian “Tree of Youth” Prolongs Longevity and Enhances Stress Resistance in *Caenorhabditis elegans*

**DOI:** 10.3390/molecules23030534

**Published:** 2018-02-27

**Authors:** Herbenya Peixoto, Mariana Roxo, Hector Koolen, Felipe da Silva, Emerson Silva, Markus Santhosh Braun, Xiaojuan Wang, Michael Wink

**Affiliations:** 1 Institute of Pharmacy and Molecular Biotechnology, Heidelberg University, INF 364, D-69120 Heidelberg, Germany; hspeixoto1@gmail.com (H.P.); marianaroxocorreia@gmail.com (M.R.); m.braun@uni-heidelberg.de (M.S.B.); wxjsz@hotmail.com (X.W.); 2 Metabolomics and Mass Spectrometry Research Group, Amazonas State University (UEA), Manaus 690065-130, Brazil; hectorkoolen@gmail.com; 3 Department of Chemistry, Federal University of Amazonas (UFAM), 6200 General Rodrigo, Manaus 69077-000, Brazil; felipesaquarema@bol.com.br; 4 Faculty of Pharmaceutical Science, Federal University of Amazonas (UFAM), 6200 General Rodrigo, Manaus 69077-000, Brazil; eslima75@gmail.com

**Keywords:** *Caenorhabditis elegans*, antioxidants, oxidative stress, aging, *Calycophyllum spruceanum*

## Abstract

The tree popularly known in Brazil as mulateiro or pau-mulato (*Calycophyllum spruceanum* (Benth.) K. Schum.) is deeply embedded in the herbal medicine of the Amazon region. Different preparations of the bark are claimed to have anti-aging, antioxidant, antimicrobial, emollient, wound healing, hemostatic, contraceptive, stimulant, and anti-diabetic properties. The current study aims to provide the first step towards a science-based evidence of the beneficial effects of *C. spruceanum* in the promotion of longevity and in the modulation of age-related markers. For this investigation, we used the model system *Caenorhabditis elegans* to evaluate in vivo antioxidant and anti-aging activity of a water extract from *C. spruceanum*. To chemically characterize the extract, HPLC MS (High Performance Liquid Chromatography Mass Spectrometry)/MS analyses were performed. Five secondary metabolites were identified in the extract, namely gardenoside, 5-hydroxymorin, cyanidin, taxifolin, and 5-hydroxy-6-methoxycoumarin-7-glucoside. *C. spruceanum* extract was able to enhance stress resistance and to extend lifespan along with attenuation of aging-associated markers in *C. elegans*. The demonstrated bioactivities apparently depend on the DAF-16/FOXO pathway. The data might support the popular claims of mulateiro as the “tree of youth”, however more studies are needed to clarify its putative benefits to human health.

## 1. Introduction

*Calycophyllum spruceanum* (Benth.) Hook. f. ex K. Schum. (syn. *Calycophyllum multiflorum* and *Eukylista spruceana*) is an Amazon native species from the family Rubiaceae found in the Amazon basin territory comprising Brazil, Bolivia, Ecuador, and Peru [1]. The tree is popularly known in Brazil by the name Mulateiro or Pau Mulato and its timber is intensively explored by the wood industry [2,3,4]. In traditional medicine, it is claimed to work as anti-aging (skin wrinkles and aging spots), antimicrobial (antibacterial, antifungal, anti-parasitic), emollient, wound healing, hemostatic, contraceptive, stimulant, and anti-diabetic [1,5,6]. Many of the medicinal indications of *C. spruceanum* are derived from the traditional knowledge of native indigenous populations of the Amazon forests [7,8].

The stem bark of *C. spruceanum* is the part of [1] the tree most commonly used in medicinal preparations. Amazonian indigenous people prepare a stem bark infusion to treat skin infections and aging; after bathing, they apply the infusion all over the body and allow it to dry. Ese Ejja, indigenous people from the Bolivian and Peruvian part of Amazon, apply poultices or compresses of stem barks to treat skin wounds [7]. The Peruvian Shipibo-Conibo tribe uses the bark against fungal skin infections [8]. Because of its broad use in the treatment of skin disorders and its characteristic annual stem bark shedding, the tree is commonly referred as “the tree of youth”.

So far, only one study has addressed the chemical composition of *C. spruceanum* bark. Zuleta et al. [9] focused on the *seco*-iridoid fraction of an ethanol extract of dried stem barks and reported three new *seco*-iridoids, namely, 7-methoxydiderroside, 6′-*O*-acetyldiderroside, and 8-*O*-tigloyldiderroside, together with loganetin, loganin, kingiside, secoxyloganin, and diderroside. Using Folin-Ciocalteu assay, Vargas et al. [10] determined the total phenolic content of an ethanol extract of stem barks as 60.2 GAE/g of sample.

Only few reports exist on the biological activities of *C. spruceanum*. Portillo et al. [11] reported a broad-spectrum antifungal activity of a dichloromethane extract from the bark against *Candida cladosporioides, Cryptococcus neoformans, Fusarium oxysporum* var. *pinaster, Microsporum gypseum, Penicillium purpurogenum, Saccharomyces cerevisiae,* and *Trichophyton mentagrophytes*, fungi associated with skin and mucosal infections. Wen et al. [8] studied the activity of an ethanolic bark extract against *T. mentagrophytes*, one of the most common etiologic agents of *tinea unguium* and *tinea pedis* [12].

Anti-parasitic activity against trypomastigote forms of *Trypanosoma cruzi* was reported by Zuleta et al. [9] for compounds that were isolated from an ethanol extract of barks, namely 7-methoxydiderroside, 6′-*O*-acetyldiderroside, secoxyloganin, and diderroside. Antioxidant activity was demonstrated through several methods (DPPH, ABTS, superoxide anion radicals, singlet oxygen, β-carotene bleaching, and murine fibroblasts). In addition, the extract was shown to inhibit horseradish peroxidase and myeloperoxidase, key enzymes involved in the acute and chronic vascular inflammatory disease [10].

Based on the well-documented and orally spread indigenous knowledge, mulateiro was incorporated in the modern herbal medicine of Brazil. Dried barks can be easily found in local traders of medicinal plants and the cosmetic industry has shown interest on the beneficial effects of *C. spruceanum* against skin disorders [13]. As *C. spruceanum* stem bark sheds completely and regenerates every year, it has a high potential for a sustainable industrial exploitation. 

In the current study, a water extract from the stem bark of *Calycophyllum spruceanum* was investigated regarding its antioxidant and anti-aging properties using the nematode *Caenorhabditis elegans*, a model organism often used in this context [14,15,16]. The investigation included the use of several mutant worm strains and uncovered important traits that are related to the molecular mechanisms triggered by the extract to perform its biological activities, which comprise enhanced resistance against oxidative stress and lifespan extension, followed by attenuation of aging markers. Additionally, we investigated the antimicrobial activity of the bark extract.

## 2. Material and Methods

### 2.1. Plant Material and Extract

*C. spruceanum* extract (CE) was obtained from 200 g stem bark purchased from a local trader in Manaus-AM (Brazil). The barks were weighted, minced, and exhaustively extracted with distilled water (5 × 1 L) at room temperature during an overall extraction period of five days. Using a rotary evaporator (Heidolph Instruments GmbH & Co. KG, Schwabach, Germany), the extract was reduced until approximately 1/4 of the initial volume at low pressure, 40 °C, and subsequently frozen at −80 °C. The frozen extract was lyophilized yielding a fine dried powder.

The plant material used in this study is deposited at the herbarium collection of IPMB (Institut für Pharmazie und Molekulare Biotechnologie, Heidelberg, Germany) under the accession number IPMB P8635.

### 2.2. DPPH

The assay was done following the method described by Blois [17] adapted to a 96-wells microplate (Greiner Bio-One GmbH, Frickenhausen, Germany). For the assay 100 μL of sample were added to 100 μL of 200 µM DPPH solution. For 30 min the plate was kept at room temperature and protected from the light; subsequently, the absorbance as measured at 517 nm. To calculate the scavenging activity, it was used the equation indicated below:

DPPH scavenging effect (%) = [(A0 − A1)/A0] × 100(1)

where, A0 = absorbance of the control and A1 = absorbance in the presence of sample. All of the measurements were performed in triplicate. The EC_50_ value was estimated by sigmoid non-linear regression and is presented in µg/mL.

### 2.3. Total Phenolic Content

The total phenolic content of the extract was done following Folin-Ciocalteu method adapted to 96-well microplate. 20 µL of sample were added to 100 µL of Folin-Ciocalteu reagent; 5 min later, 80 µL of sodium carbonate (7.5% solution) were added to each well. For 2 h, the plate was kept at room temperature and protected from the light; subsequently, the absorbance as measured at 750 nm. All of the measurements were carried out in triplicate and at least three times. The phenolic content is expressed as gallic acid equivalents (GAE/g of sample).

### 2.4. Chemical Characterization

HPLC analyses were performed using Accela liquid chromatography system (Thermo, Waltham, MA, USA) equipped with a binary pump system (Accela 600) and a luna-C18 column (150 mm × 4.6 mm i.d., 5 μm particle size) (Phenomenex, Torrance, CA, USA). Solvent A was water and B was acetonitrile. The gradient elution at 28 °C was as follows: 0–24 min, 20–100% (*v*/*v*) B; and, 100% B isocratic; 24–40 min. For this analytical method, the flow rate was 1.0 mL/min and the sample volume injected was 25 μL (sample extract at 0.5 mg/mL). The HPLC system was coupled to a mass spectrometer (MS): TSQ Quantum Access triple quadrupole (Thermo, Waltham, MA, USA) using electrospray ionization (ESI), operating in the positive mode. The data were acquired in scan mode at *m*/*z* range of 100 to 1200. The ionization source working conditions were as follows: Capillary voltage, 4.5 kV; Source temperature, 250 °C; Cone gas flow rate, 70 L/h; Desolvation gas flow rate, 600 L/h; and desolvation temperature, 350 °C. Nitrogen (>99% purity) and argon (99% purity) were used as nebulizing and collision (product ion scan, MS/MS) gases, respectively. Data acquisition was carried out with Xcalibur v 2.7 software (Thermo, Waltham, MA, USA). 

### 2.5. C. elegans Strains and Maintenance

The nematodes (N2 (wt)), CF1038 (daf-16(mu86)), GR1307 (daf-16(mgDf50)), CF1553 (muIs84 [(pAD76) sod-3p::GFP + rol-6]), TJ375 (gpIs1[hsp-16-2::GFP]), and BA17 (fem-1(hc17) IV)) were cultivated on nematode growth media (NGM) plates inoculated with living *E. coli* OP50 as a food source and incubated at 20 °C, except when referred. Age synchronized worms were obtained by treating gravid adults with a lysis solution (5 M NaOH and 5% NaOCl) for 5 min. The lysate was pelleted by centrifugation (1200 rpm, 1 min) and the eggs were separated from the debris by density gradient centrifugation: 5 mL sucrose solution (60%) and 5 mL sterile water (4 min, 1200 rmp). To wash out the sucrose, the upper layer containing the eggs was transferred to a fresh tube added of 5 mL sterile water and centrifuged (1 min, 1200 rpm). The collected eggs were allowed to hatch in M9 buffer [18].

The *C. elegans* strains and *E. coli* OP50 used in the present work were purchased from the Caenorhabditis Genetics Center (CGC), University of Minnesota, Minneapolis, MN, USA.

### 2.6. Survival Assay under Oxidative Stress 

Age synchronized worms (N2, CF1038 and GR1307) grown in S-medium were separated into groups of 75 worms at L1 larval stage and treated with CE for 48 h, except for the control. Subsequently, the groups were exposed to 80 µM of the pro-oxidant naphthoquinone juglone (Sigma-Aldrich GmbH, Steinheim, Germany) and 24 h later the number of dead and live worms was scored. The worms were considered dead when they did not respond to gentle touch with a platinum wire. The result is presented as mean survival rate and compared by one-way ANOVA followed by Bonferroni correction.

### 2.7. Intracellular ROS Accumulation 

Age synchronized N2 worms (L1 stage, grown in S-medium) were sorted into groups and were treated with CE for 48 h, except the control group. Afterwards, the worms were washed with M9 buffer and incubated for 1 h at 20 °C with 50 µM CM-H2DCFDA (Fluka Chemie GmbH, Buchs, Switzerland), an indicator of ROS. Washed again with M9 and mounted on a glass slide with a drop of 10 mM sodium azide for paralysis. Live images were captured of at least 30 worms per group, using a fluorescence microscope (λex 480/20 nm; λem 510/38 nm) (BIOREVO BZ-9000, Keyence Deutschland GmbH, Neu-Isenburg, Germany). The relative fluorescence of the whole body was determined densitometrically using Image J version 1.48 (National Institute of Health, Bethesda, MD, USA). The result is presented as mean fluorescence intensity (mean ± SEM) and compared by one-way ANOVA followed by Bonferroni correction.

### 2.8. Quantification of Sod-3::GFP and Hsp-16::GFP Expression

Age synchronized worms (CF1553, L1 stage, grown in S media) carrying a GFP reporter fused with *sod*-3 were treated with CE for 48 h, except the control group and submitted to fluorescence microscopy, as described above. The relative fluorescence of the posterior intestine was determined densitometrically using Image J. The results are presented as mean fluorescence intensity (mean ± SEM) and compared by one-way ANOVA followed by Bonferroni correction.

Age synchronized worms (TJ375, L4 stage, grown in S-medium) carrying a GFP reporter fused with *hsp*-16.2 were sorted into populations and treated with CE for 48 h, except for the control group. Subsequently, 20 µM juglone was added to the medium and 24 h later the worms were submitted to fluorescence microscopy. The relative fluorescence of the head of the worms was determined densitometrically using Image J. The results are presented as mean fluorescence intensity (mean ± SEM) and compared by one-way ANOVA followed by Bonferroni correction.

### 2.9. Longevity Assay

Age synchronized worms (BA17, grown in S-medium, 25 °C, at day 1 of adulthood) were sorted and treated with CE, except the control group. Live worms were counted and transferred every second day to fresh medium supplemented according to their treatment group. The individuals exhibiting internally hatched progeny or extruded gonads were scored as censored worms and excluded from the assay. Dead worms, those that did not respond to a gentle touch with the platinum wire, were scored and removed from the assay. The results are presented as percentage of survival and the statistical significance was determined by Log-rank (Mantel-Cox) tests, followed by Gehan-Breslow-Wilcoxon Test.

### 2.10. Pharyngeal Pumping Rate

Age synchronized worms (N2, day 1 of adulthood, grown in S-medium) were sorted and placed on NGM agar plates seeded with *E. coli* OP50. The bacterial lawn was supplemented with CE in the treated groups. The adults were daily transferred to separate them from their progeny; after day 5 of adulthood the transfer started to be done on the day before the analyses of the pumping activity, which were taken on day 5 and 10 of adulthood. To score the pumping rate each worm was observed for 1 min when crawling on the bacterial lawn using a stereoscope. Each group contained a minimum of 10 worms. The results are presented as pumps/min (mean ± SEM) and compared by two-way ANOVA followed by Bonferroni (post-hoc).

### 2.11. Body Length and Brood Size

The body length was measured at day 1 of adulthood. N2 (wt) were treated with CE at L3/L4 larval stage and live images were captured in bright field microscopy from at least 30 worms using a 10× objective lens. The results are presented as body length in µm (mean ± SEM) and compared by one-way ANOVA followed by Bonferroni (post-hoc).

To measure the brood size, age synchronized worms (N2, L4 stage, grown in S-medium) were sorted and placed individually on NGM agar plates seeded with *E. coli* OP50. From day 1 of adulthood, the adults started to be transferred daily to fresh plates, in order to allow for the counting of eggs and their separation from the progeny. The procedure was repeated for five days. The mean brood size was compared by two-way ANOVA followed by Bonferroni (post-hoc). The bacterial lawn was supplemented with CE in the treated groups.

### 2.12. Carbonyl Content

Age synchronized N2 (wild type) worms, cultured at 20 °C in S-medium inoculated with living *E. coli* OP50, were separated at day 1 of adulthood into groups and treated with CE, except the control group. At day 5 of adulthood, the worms were lysed with RIPA buffer containing DNase I (1.5 µL) to obtain a crude protein extract. The protein concentration of the crude extract was measured by BCA assay and adjusted to 0.5 mg/mL. The carbonyl content was assessed through DNPH assay, following the instruction of the manufacturer (Sigma-Aldrich GmbH, St. Louis, MO, USA).

### 2.13. Antimicrobial Activity

Susceptibility of the Gram-negative bacteria *Escherichia coli* strain OP50 was assessed by means of well diffusion test, according to CLSI (2014) with minor modifications [19]. Briefly, bacteria were grown on Müller-Hinton agar (MHA). A cell suspension was adjusted to 0.5 McFarland standard. Bacteria was evenly spread on MHA. Wells with 6 mm in diameter were punched out and loaded with 70 µL of 10 mg/mL sample dissolved in sterile water. Ampicillin and Ciprofloxacin (256 µg/mL) were used as positive controls. Diameters of the zones of inhibition (ZI) were read after incubation at 35 °C for 24 h. The test was repeated three times. All of the microorganisms were provided by the Department of Infectious Diseases, Medical Microbiology and Hygiene, Heidelberg University, Heidelberg, Germany.

### 2.14. Statistical Analyses

The statistical analyses were done using the software Graphpad Prism for Windows, Version 6.01 (GraphPad Software, La Jolla, CA, USA). The results were compared by one-way ANOVA followed by Bonferroni’s correction (post hoc) or two-way ANOVA when appropriate. All of the assays were repeated at least three times.

## 3. Results

### 3.1. Antioxidant Activity In Vitro and Total Phenolic Content

Anti-radical activity of CE was assessed through determination of its capacity to scavenge free DPPH radicals. Our results demonstrated a powerful antioxidant capacity of CE, comparable to that of standard antioxidants like vitamin C and the polyphenol Epigallocatechin gallate (EGCG). The corresponding IC_50_ values are shown in Table 1. The obtained result correlates with the high phenolic content revealed by Folin-Ciocalteu method (1120 GAE/g extract).

### 3.2. Chemical Characterization

**Figure 1:**
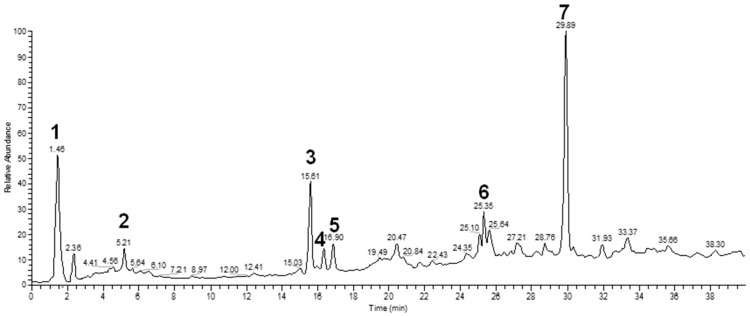
Chemical profile of *Calycophyllum spruceanum* water extract. Seven major peaks were identified by MS/MS analyses.

The water extract was phytochemically characterized by HPLC MS/MS using Electrospray Ionization (ESI). Five compounds could be identified among the seven major peaks that were observed in the HPLC profile of CE extract (Figure 1).

When comparing the retention time and mass spectral data of the selected peaks with data available in literature it was possible to identify them as gardenoside, 5-hydroxymorin, cyanidin, taxifolin, and 5-hydroxy-6-methoxycoumarin-7-glucoside. The peaks 1 and 4 could not be identified by this approach (Table 2).

### 3.3. In Vivo Antioxidant Activity

**Figure 2:**
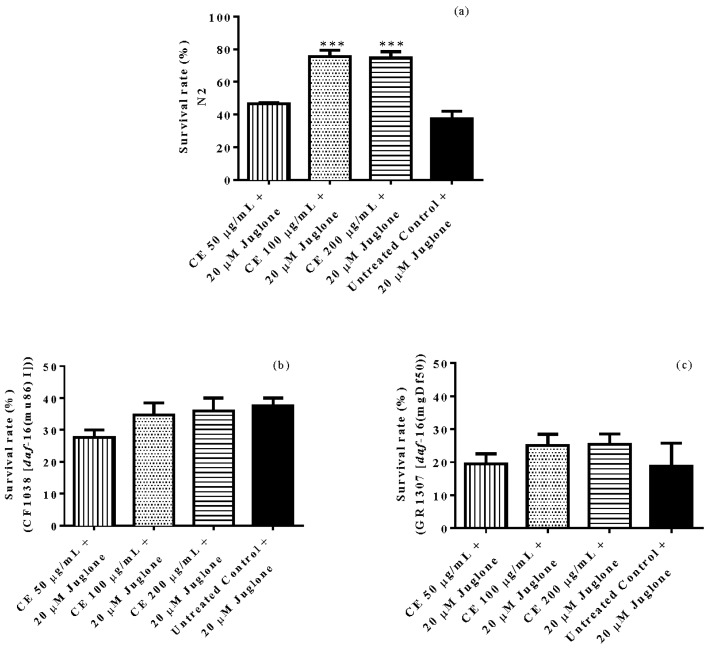
Survival of nematodes after juglone-induced oxidative stress. Survival rate of N2 worms was significantly enhanced in the groups treated with *C. spruceanum* extract (CE) (**a**). However, the survival rate of DAF-16 mutants CF1038 [*daf*-16(mu86) I]) (**b**) and GR1307 [*daf*-16(mgDf50) I] (**c**) was not different between the groups. Each bar represents the mean ± SEM from three independent assays. *** *p* < 0.001, compared to the untreated control by one-way ANOVA followed by Bonferroni (post-hoc).

In vivo antioxidant activity was assessed comparing the survival rate of treated and untreated N2 (wt) worms after juglone-induced oxidative stress. The results indicate a significantly higher survival rate among CE treated worms when compared with untreated worms. The highest tested concentration, 200 µg/mL CE, resulted in 77% of live individuals after juglone treatment while in the control group this score was up to 37% (adjusted *p*-value = 0.0002). However, the survival rate of CE treated and untreated worms from the mutant strains CF1038 ([*daf*-16(mu86)I]) and GR1307 ([*daf*-16(mgDf50)] was not significantly different, indicating the involvement of the transcription factor DAF-16 (Figure 2).

### 3.4. Quantification of Intracellular ROS Accumulation

**Figure 3:**
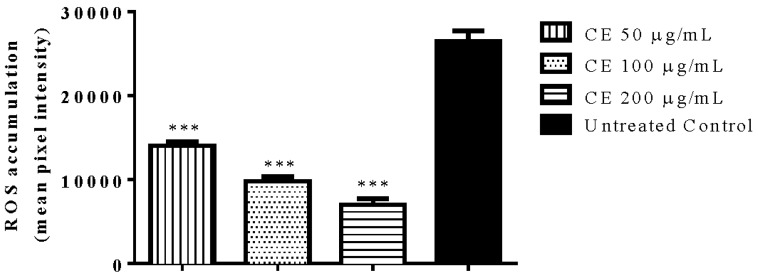
Quantification of intracellular ROS in N2 worms using DCFDA. Worms treated with *C. spruceanum* extract (CE) showed lower levels of ROS compared to the control group. Data are presented as mean pixel intensity ± SEM (*n* = 40, replicated 3 times). *** *p* < 0.001, compared to the untreated control by one-way ANOVA followed by Bonferroni (post-hoc).

The intracellular ROS accumulation under physiological conditions was assessed in N2 (wt) worms. The results indicated a significant decrease in ROS accumulation among CE treated worms compared with the untreated control group. The decrease was up to 80% when the worms were treated with 200 µg/mL CE (adjusted *p*-value < 0.0001) (Figure 3).

### 3.5. Quantification of Gene Expression of Sod-3 and Hsp-16.2

**Figure 4:**
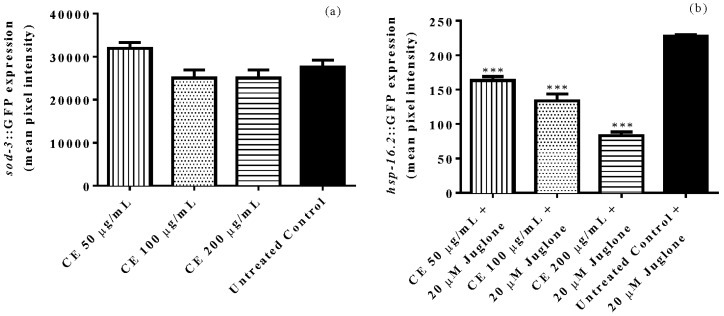
Quantification of the expression of stress response genes in mutant worms through fluorescence microscopy. Mutants CF1553 [(pAD76)*sod*-3::GFP + rol-6] treated with *C. spruceanum* extract (CE) exhibited no difference in sod-3::GFP expression when compared with the untreated control group (**a**). After 20 µM juglone exposure, mutants TJ375 [*hsp*-16.2::GFP(gplsI)] treated with CE exhibited lower expression of hsp-16::GFP compared with the untreated control (**b**). The results are presented as mean ± SEM from three independent assays. *** *p* < 0.001, as compared to the untreated control by one-way ANOVA followed by Bonferroni (post-hoc).

The expression of *sod*-3 was assessed using mutant worms of the strain CF1553, which has *sod*-3 fused with GFP reporter. By analyses of the emitted fluorescence intensity no significant difference in *sod*-3::GFP expression was observed among CE treated worms when compared with the untreated control (Figure 4a).

The expression of the stress marker *hsp*-16.2 was investigated using the mutant strain TJ375 that has *hsp*-16.2 fused with GFP. After mild oxidative stress induced by 20 µM juglone, we observed a significantly lower fluorescence intensity among CE treated worms when compared with untreated control worms. The decrease was up to 63% at 200 µg/mL CE (adjusted *p*-value < 0.0001) (Figure 4b).

### 3.6. Longevity Study

**Figure 5:**
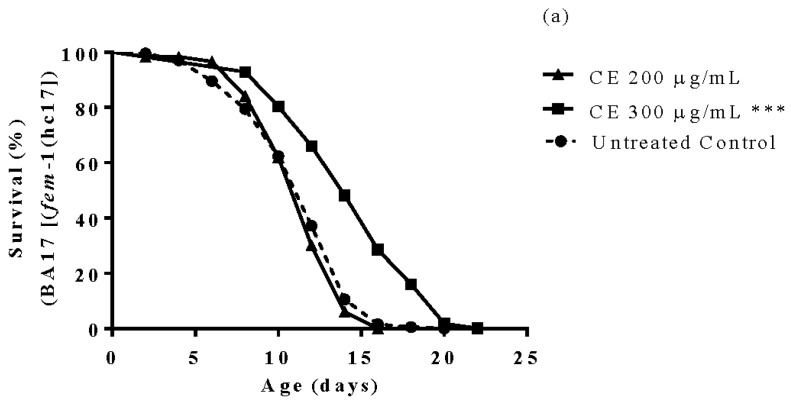

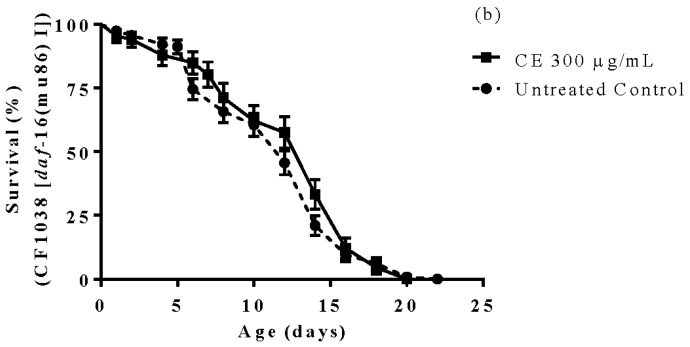
Longevity of nematodes after treatment with *C. spruceanum* extract (CE). Worms (BA17) treated with CE 300 µg/mL lived significantly longer compared to control group (**a**). Worms CF1038 treated with CE or untreated exhibited the same lifespan (**b**). The results are presented as percentage of survival and the statistical significance determined by Log-rank (Mantel-Cox) tests followed by Gehan-Breslow-Wilcoxon Test. *** *p* < 0.0001. as compared to the untreated control by one-way ANOVA followed by Bonferroni (post-hoc).

Worms treated with 300 µg/mL CE exhibited a lifespan extension of approximately 16% when compared with the untreated control worms (BA17 [fem-1(hc17)]) cultured under the same conditions (*p*-value < 0.0001). However, when tested in worms from the strain CF1038, which carry a *daf*-16.2 null mutation, the lifespan extension effect was not observed (Figure 5).

### 3.7. Pharyngeal Pumping Rate

**Figure 6:**
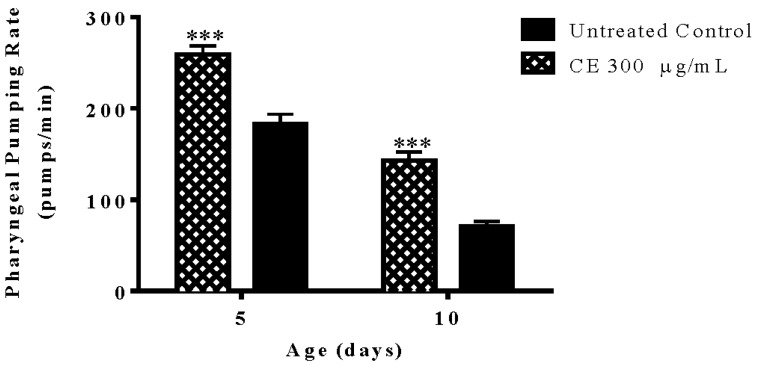
Pharyngeal pumping rate after treatment with *C. spruceanum* extract (CE). The treatment of wild type worms with 300 μg/mL CE significantly attenuated the age-associated decline in the pharyngeal muscle function. Data are presented as mean ± SEM. *** *p* < 0.001 related to the control by a two-way ANOVA.

The pumping activity was scored in CE treated and untreated N2 (wt) worms at day 5 and 10 of adulthood. The results obtained indicate that CE is able to significantly attenuate the decline in pharynx muscle function, which accompanies the aging process. The pumping activity of the pharynx scored in CE treated group at the last day of assessment was 58% higher than the untreated control group (*p*-value < 0.01) (Figure 6).

### 3.8. Brood Size and Body Length

**Figure 7:**
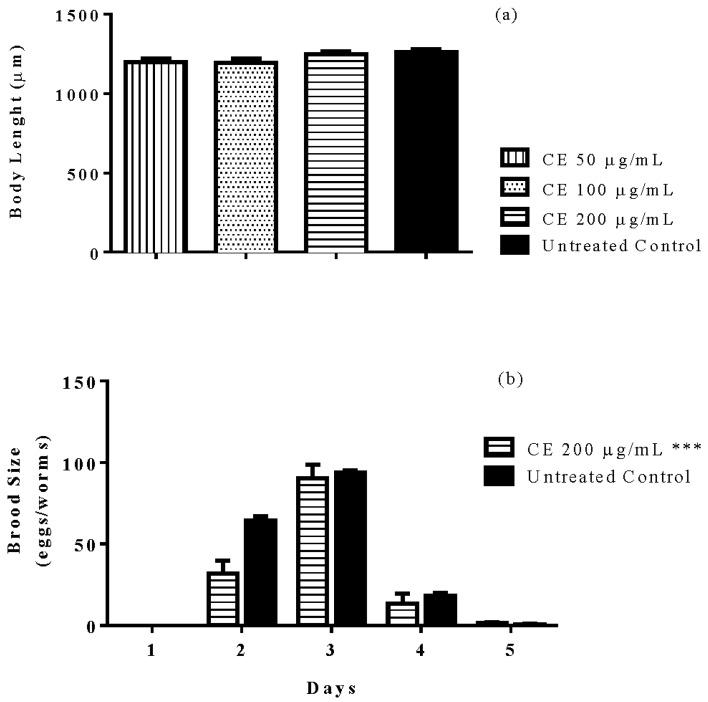
Brood size and body length of worms after treatment with *C. spruceanum* extract (CE). The treatment of N2 (wt) worms did not impair the body development (**a**), but significantly decreased the brood size of treated worms (**b**). Data are presented as mean ± SEM. *** *p* < 0.0001 related to the control by two-way ANOVA.

CE treatment affected the fertility rate of N2 (wt) worms; analyses of the brood size revealed a significantly lower mean number of eggs laid per day among worms treated with 300 µg/mL CE when compared with the untreated control group. The body length, a marker of development and caloric restriction, did not change after CE treatment (Figure 7).

### 3.9. Protein Carbonyl Content

Worms N2 (wt) treated with 300 µg/mL CE exhibited a significantly lower level of protein oxidation (untreated worms: 29.48 nmol carbonyl/mg of protein; CE treated worms: 11.43 nmol carbonyl/mg of protein). The protein carbonyl content, assessed through DNPH assay, was up to 40% lower when compared with the untreated control group (*p*-value = 0.01).

### 3.10. Antimicrobial Activity

CE was tested against *E. coli* OP50 and no microbicide effect was observed, this result excludes caloric restriction as the promoter of lifespan extension observed in CE treated groups.

## 4. Discussion

Previous studies on the phytochemical composition of the stem bark of *C. spruceanum* revealed the presence of iridoid glucosides [9]. In the current study, the iridoid gardenoside, the flavonoids 5-hydroxymorin and taxifolin, the anthocyanin cyanidin and the coumarin 5-hydroxy-6-methoxycoumarin-glucoside were identified. The polyphenols identified correlate with the high phenolic content indicated by Folin-Ciocalteu and the high antioxidant activity demonstrated in vitro by DPPH assay.

When considering the well-known activity of phenolic compounds in conferring stress resistance [20], the antioxidant activity of CE extract was investigated using *C. elegans* as a model organism. N2 (wild type) worms treated with CE exhibited an enhanced survival rate after juglone-induced oxidative stress in comparison with untreated worms. In agreement with this result we found lower level of cellular reactive oxygen species (ROS) in worms treated with the extract; correspondingly, the protein carbonyl content, which is a marker of ROS induced protein oxidation, was significantly lower in CE treated worms. In agreement with these findings, the expression of *hsp*-16.2, triggered by juglone exposure, was significantly attenuated after CE treatment. *Hsp*-16.2 is a small heat shock protein whose expression is induced in *C. elegans* when the worm is facing harsh environmental conditions, such as overheating and oxidative damage [21,22], the low expression of *hsp*-16.2::GFP observed in CE treated worms supports the assumption that the extract is an effective antioxidant in vivo.

Phenolic rich plant extracts like those obtained from *Aspalathus linearis* (rooibos tea), *Camellia sinensis* (green tea), and *Paullinia cupana* (guarana) are known to extend lifespan in *C. elegans* [14,15,23]. The lifespan extension promoted by those extracts are due to the capacity of their secondary metabolites to modulate molecular pathways directly involved in the control of aging and longevity, as well as their antioxidant properties in vivo [20,23,24,25]. Similarly, the water extract from *C. spruceanum* was able to extend the lifespan of feminized *C. elegans* mutants (BA17) by 16%. This result appears to be correlated with the phenolic profile of *C. spruceanum* and its antioxidant capacity in vivo. Moreover, *C. spruceanum* lifespan extension was accompanied by attenuation of age-related muscle function decline, assessed through the pharynx pumping activity in early and late adulthood, which is an important aging marker [26]. This result also indicates that the ability of the worms to feed on bacteria was not impaired by CE treatment and consequently the caloric intake was not affected. Furthermore, no antimicrobial effect of CE was seen against *E. coli* OP50; the data supports the assumption that the lifespan extension promoted by *C. spruceanum* extract is not due to induction of caloric restriction, since the feeding behavior and the food source availability was not impaired by the treatment.

Longevity and aging have been demonstrated to be under genetic control, but are sensitive to environmental stimuli and stochastic factors [27,28,29]. Data available in literature show that polyphenols can modulate signaling pathways that are involved in the control of aging and longevity, attenuating aging-markers and extending lifespan. Resveratrol, a well-studied phenol from grapes and other plants, has been suggested to extend lifespan in *C. elegans* through the modulation of SIRT-1 [30,31], although it was only possible to see lifespan extension from resveratrol in worms under stress condition [32]. EGCG promotes lifespan extension and upregulation of *daf-16* and *skn-1* [33]; the DAF-16 transcription factor, homologue of the mammalian FOXO is a key modulator of the stress resistance, longevity, and other important cellular functions [34,35,36]. In the current study, when CE was tested in DAF-16 mutant worms no significant effect was observed in survival rate, ROS accumulation and lifespan, conversely to the results observed with N2 (wt) worms. These findings support the assertion that CE extract enhances stress resistance, extends lifespan and attenuates aging in a DAF-16/FOXO dependent manner.

Worms treated with CE did not show impairment in the development rate assessed through the measurement of the body length, but, regarding the fertility rate, we observed a significant difference in the brood size of treated worms. Noteworthy, mutations that affect DAF-2 or other components of the insulin signaling pathway (IIS) like AGE-1 also promoted lifespan extension, enhanced stress resistance and decrease in fertility rate. The main transcription factor transducing the signals from IIS is DAF-16/FOXO [37]. Apparently, CE extract works through a similar mechanism, yet further studies are needed to unveil the pathway up stream of DAF-16/FOXO, which might be triggered by CE.

In summary, CE water extract exhibited impressive antioxidant activity in vivo and in vitro. The extract possibly works though DAF-16/FOXO molecular pathway to extend lifespan and enhance stress resistance, thus improving survival after induced oxidative stress, decreasing cellular ROS accumulation and expression of *hsp*-16.2. The lifespan extension elicited by CE is followed by attenuation of the age-related muscle function decline. Additional studies are needed to unveil the molecular mechanisms evoked by CE to perform its biological activities. Nonetheless, the presented work supports the indigenous-based anti-aging claims of *C. spruceanum*. The assessment of its safety profile for human consumption is strongly suggested.

## Figures and Tables

**Table 1:** Antiradical activity of tested samples according to DPPH assay.

Sample	IC_50_ *
CE	3.00 ± 0.07
EGCG	1.03 ± 0.06
Vitamin C	±0.04

* µg/mL.

**Table 2:** Identification of secondary metabolites in *Calycophyllum spruceanum* water extract by LC MS/MS using ESI.

Peak	R_t_	*m*/*z* [M + H]^+^	Fragment Ions	Tentative of Identification	Chemical Structure
1	1.48	329	311, 293, 275, 251, 209	unkown	-
2	5.21	405	387, 355, 323, 193, 167	gardenoside	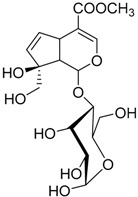
3	15.61	319	301, 273, 259	5-hydroxymorin	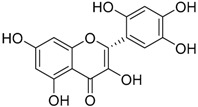
4	10.64	287	269	unknown	-
5	11.52	321	285, 257, 247, 221	cyanidin	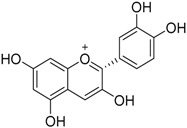
6	11.78	305	287, 259, 221, 191, 175, 163	taxifolin	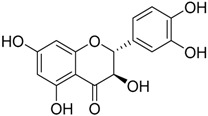
7	12.01	371	259, 241, 147, 129	5-hydroxy-6-methoxycoumarin 7-glucoside	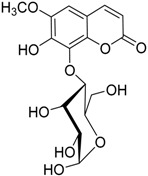

## References

[1] Duke J.A., Godwin M., Ottesen A. (2009). Duke’s Handbook of Medicinal Plants of Latin America.

[2] Kvist L.P., Andersen M.K., Stagegaard J., Hesselsøe M., Llapapasca C. (2001). Extraction from woody forest plants in flood plain communities in amazonian peru: Use, choice, evaluation and conservation status of resources. For. Ecol. Manag..

[3] Weber J.C., Montes C.S., Ugarte J., Simons T. (2009). Phenotypic selection of *Calycophyllum spruceanum* on farms in the Peruvian amazon: Evaluating a low-intensity selection strategy. Silvae Genet..

[4] Tauchen J., Lojka B., Hlasna-Cepkova P., Svobodova E., Dvorakova Z., Rollo A. (2011). Morphological and genetic diversity of *Calycophyllum spruceanum* (Benth) k. Schum (Rubiaceae) in Peruvian amazon. Agric. Trop. Subtrop..

[5] Brioso P.S.T. (2012). Chanker in *Calycophyllum spruceanum* in Rio de Janeiro state. Summa Phytopathol..

[6] Santos A., Ribeiro-Oliveira J., Carvalho C. (2016). On the botany, ethnopharmacology, and chemistry of *Calycophyllum spruceanum* (Benth.) Hook. F. Ex k. Schum. Rev. Bras. Plantas Med..

[7] Alexiades M.N. (1999). Ethnobotany of the Ese Eja: Plants, Health, and Change in an Amazonian Society. Ph.D. Thesis.

[8] Wen L., Haddad M., Fernández I., Espinoza G., Ruiz C., Neyra E., Bustamante B., Rojas R. (2011). Actividad antifúngica de cuatro plantas usadas en la medicina tradicional peruana: Aislamiento de 3′-formil-2′, 4′, 6′-trihidroxidihidrochalcona, principio activo de *Psidium acutangulum*. Revista de la Sociedad Química del Perú.

[9] Zuleta L.M.C., Cavalheiro A.J., Silva D.H.S., Furlan M., Young M.C.M., Albuquerque S., Castro-Gamboa I., da Silva Bolzani V. (2003). Seco-iridoids from *Calycophyllum spruceanum* (Rubiaceae). Phytochemistry.

[10] de Vargas F.S., Almeida P.D., de Boleti A.P.A., Pereira M.M., de Souza T.P., de Vasconcellos M.C., Nunez C.V., Pohlit A.M., Lima E.S. (2016). Antioxidant activity and peroxidase inhibition of amazonian plants extracts traditionally used as anti-inflammatory. BMC Complement. Altern. Med..

[11] Portillo A., Vila R., Freixa B., Adzet T., Cañigueral S. (2001). Antifungal activity of Paraguayan plants used in traditional medicine. J. Ethnopharmacol..

[12] Ameen M. (2010). Epidemiology of superficial fungal infections. Clin. Dermatol..

[13] Funasaki M., Barroso H.d.S., Fernandes V.L.A., Menezes I.S. (2016). Amazon rainforest cosmetics: Chemical approach for quality control. Química Nova.

[14] Chen W., Sudji I.R., Wang E., Joubert E., van Wyk B.-E., Wink M. (2013). Ameliorative effect of aspalathin from rooibos (*Aspalathus linearis*) on acute oxidative stress in *Caenorhabditis elegans*. Phytomedicine.

[15] Abbas S., Wink M. (2014). Green tea extract induces the resistance of *Caenorhabditis elegans* against oxidative stress. Antioxidants.

[16] Peixoto H., Roxo M., Krstin S., Röhrig T., Richling E., Wink M. (2016). An anthocyanin-rich extract of acai (*Euterpe precatoria* Mart.) increases stress resistance and retards aging-related markers in *Caenorhabditis elegans*. J. Agric. Food Chem..

[17] Blois M.S. (1958). Antioxidant determinations by the use of a stable free radical. Nature.

[18] Stiernagle T. Maintenance of C. Elegans.

[19] Ashour M.L., El-Readi M.Z., Hamoud R., Eid S.Y., El Ahmady S.H., Nibret E., Herrmann F., Youns M., Tahrani A., Kaufmann D. (2014). Anti-infective and cytotoxic properties of *Bupleurum marginatum*. Chin. Med..

[20] van Wyk B.-E., Wink M. (2015). Phytomedicines, Herbal Drugs, and Poisons.

[21] Strayer A., Wu Z., Christen Y., Link C.D., Luo Y. (2003). Expression of the small heat-shock protein hsp 16–2 in *Caenorhabditis elegans* is suppressed by *Ginkgo biloba* extract EGb 761. FASEB J..

[22] Swindell W.R. (2009). Heat shock proteins in long-lived worms and mice with insulin/insulin-like signaling mutations. Aging.

[23] Peixoto H., Roxo M., Röhrig T., Richling E., Wang X., Wink M. (2017). Anti-aging and antioxidant potential of *Paullinia cupana* var. *sorbilis*: Findings in *Caenorhabditis elegans* indicate a new utilization for roasted seeds of guarana. Medicines.

[24] Scalbert A., Johnson I.T., Saltmarsh M. (2005). Polyphenols: Antioxidants and beyond. Am. J. Clin. Nutr..

[25] Pant A., Pandey R. (2015). Bioactive phytomolecules and aging in *Caenorhabditis elegans*. Healthy Aging Res..

[26] Rothman J.H., Singson A. (2012). Caenorhabditis Elegans: Cell Biology and Physiology.

[27] Candore G., Balistreri C.R., Listi F., Grimaldi M.P., Vasto S., Colonna-Romano G., Franceschi C., Lio D., Caselli G., Caruso C. (2006). Immunogenetics, gender, and longevity. Ann. N. Y. Acad. Sci..

[28] Goldsmith T.C. (2006). The Evolution of Aging: How New Theories will Change the Future of Medicine.

[29] Harman D. (1972). The biologic clock: The mitochondria?. JAGS.

[30] Morselli E., Maiuri M., Markaki M., Megalou E., Pasparaki A., Palikaras K., Criollo A., Galluzzi L., Malik S., Vitale I. (2010). Caloric restriction and resveratrol promote longevity through the sirtuin-1-dependent induction of autophagy. Cell Death Dis..

[31] Borra M.T., Smith B.C., Denu J.M. (2005). Mechanism of human sirt1 activation by resveratrol. Int. J. Biol. Chem..

[32] Chen W., Rezaizadehnajafi L., Wink M. (2013). Influence of resveratrol on oxidative stress resistance and life span in *Caenorhabditis elegans*. J. Pharm. Pharmacol..

[33] Zhang L., Jie G., Zhang J., Zhao B. (2009). Significant longevity-extending effects of egcg on *Caenorhabditis elegans* under stress. Free Radic. Biol. Med..

[34] Barbieri M., Bonafè M., Franceschi C., Paolisso G. (2003). Insulin/igf-i-signaling pathway: An evolutionarily conserved mechanism of longevity from yeast to humans. Am. J. Physiol. Endocrinol. Metab..

[35] Baumeister R., Schaffitzel E., Hertweck M. (2006). Endocrine signaling in *Caenorhabditis elegans* controls stress response and longevity. J. Endocrinol..

[36] Kenyon C., Chang J., Gensch E., Rudner A., Tabtiang R.A. (1993). *C. Elegans* mutant that lives twice as long as wild type. Nature.

[37] Tissenbaum H.A., Ruvkun G. (1998). An insulin-like signaling pathway affects both longevity and reproduction in *Caenorhabditis elegans*. Genetics.

